# First Description of Oral Microbiota in Domestic Cats Affected by Oral Squamous Cell Carcinoma

**DOI:** 10.3390/pathogens15020207

**Published:** 2026-02-12

**Authors:** Jamie G. Anderson, Elisa Scarsella, Maria Soltero-Rivera, Stephanie Goldschmidt, Connie A. Rojas, Holly H. Ganz

**Affiliations:** 1 Department of Oral Medicine, Penn Dental Medicine, Philadelphia, PA 19104, USA; jgadvm@gmail.com; 2 Animal Biome, Oakland, CA 94609, USA; elisa@animalbiome.com (E.S.); connie@animalbiome.com (C.A.R.); 3 Department of Agricultural, Food, Environmental and Animal Sciences, University of Udine, 33100 Udine, Italy; 4 Department of Surgical and Radiological Sciences, School of Veterinary Medicine, University of California, Davis, CA 95616, USA; msoltero@ucdavis.edu (M.S.-R.); sgoldschmidt@ucdavis.edu (S.G.)

**Keywords:** 16S rRNA gene sequencing, biopsy, cats, oral microbiota, oral swab, oral squamous cell carcinoma

## Abstract

Oral squamous cell carcinoma (OSCC) accounts for the majority of feline oral neoplasms and carries a poor prognosis; however, the oral microbiome in affected cats remains poorly characterized. This study aimed to preliminarily describe the oral bacterial communities of cats with OSCC and compare them with those of clinically healthy cats using DNA amplicon sequencing. Oral swabs were collected from cats with OSCC, including tumor surfaces, tumor cut surfaces, and clinically normal mucosa distant from the tumor (*n* = 20 total samples), and from the gingival margin of healthy cats (*n* = 12). DNA was extracted and full-length 16S rRNA gene sequencing was performed to assess microbial composition and diversity. Cats with OSCC exhibited significant alterations in oral microbiota compared with healthy controls, including reduced alpha diversity, distinct beta-diversity clustering, and consistent taxonomic shifts. Healthy cats displayed a relatively conserved core microbiome dominated by *Porphyromonas* spp., *Bacteroides*, Pasteurellaceae, *Helcococcus*, and *Moraxella*. In contrast, OSCC-associated samples showed increased relative abundances of anaerobic and disease-associated taxa, including *Filifactor villosus*, *Bacteroides pyogenes*, *Odoribacter denticanis*, *Porphyromonas circumdentaria*, and members of the Pasteurellaceae. These findings provide the first description of the oral microbiota associated with feline OSCC and demonstrate exploratory microbial differences between health and disease.

## 1. Introduction

Oral neoplasms represent approximately 3% of all tumors in domestic cats [1]. Despite the relative rarity, these represent a clinically challenging disease process as the treatment options are often limited by the stage at which the cancer is detected and the associated prognosis is grave. Many papers suggest that feline oral squamous cell carcinoma (OSCC) could be an animal model for human head and neck cancer [2]. However, mechanistic gaps in knowledge on the pathogenesis of feline OSCC, such as the role of changes in the oral microbiota, have not been reported, limiting our understanding of the strength of feline OSCC as a spontaneous model of disease [3].

Head and neck SCC in humans is the eighth most common cancer universally and great strides have been made in its pathogenic understanding [4,5]. Smoking, alcohol consumption and Papilloma virus (PV) infection are known risk factors of oral cancer [6], although PV has controversial causality [7]. Further, dysbiosis in the oral microbiota is increasingly recognized as a co-morbidity factor in OSCC, and analysis of the microbial profiles reveals significant differences compared to healthy individuals [8,9]. Crosstalk between the oral microbiota, mucosal immunity, and the epithelial barrier regulates oral mucosal disease pathogenesis in mucosal disease and cancer [10]. Silent shifts in the oral microbiota have been implicated in promoting carcinogenesis [11] and promoting oncogenesis [12,13]. Characterization of the feline oral microbiome in OSCC and in health provides a foundation for further study of the microbiome relative to novel diagnostic markers and treatment that could be evaluated in cats as a spontaneous model of disease and benefit both species.

When investigating the oral microbiota in humans, a few terms warrant definition. The term “oral microbiome” pertains to microorganisms that inhabit the oral cavity and is separated into two categories, the core microbiome and the variable microbiome. The term “core microbiome” refers to the predominant microbial species observed in diverse anatomical locations of the human body in all individuals who exhibit sound health [14]. Distinctly, the “variable microbiome” refers to microorganisms that are unique to each individual and are influenced by factors such as the individual’s genetic composition, lifestyle, immune system, physiology, pathobiology, and environment [15,16]. Feline oral ecology could benefit from these definitions. Dysbiosis commonly describes compositional (core, variable or both) and functional alterations in the microbiota that are driven by a set of environmental and host-related factors that perturb the microbial ecosystem to an extent that exceeds its resistance and resilience capabilities [17]. A linkage between the oral microbiome and Head and Neck Squamous Cell Carcinoma (HNSCC) in people has been reported in previous studies [18,19,20,21,22]. For example, bacteria like *Streptococcus* spp., *Peptostreptococcus* spp., *Prevotella* spp., *Porphyromonas gingivalis*, and *Capnocytophaga gingivalis* are strongly associated with OSCC. *Fusobacterium*, *Clostridium*, *Enterobacteriaceae*, *Veillonella*, *Actinomyces*, and *Haemophilus* are also associated with oral cancer and other epithelial precursor lesions [12]. Additionally, high counts of *Capnocytophaga gingivalis*, *Prevotella melaninogenica*, and *Streptococcus mitis* were observed in the saliva of oral cancer patients when compared to non-cancer patients, suggesting that these three species could be used as a diagnostic indicator for OSCC, predicting 80% of cancer cases [23].

Pathogenesis, prognosis, and targeted therapeutics have become three critical arms in the scientific advancement of treating oral neoplasms. Additional biomarkers of disease are emerging as possible diagnostic tools. Critical are the current metagenomic studies on the oral microbiome in HNSCC in people [6,12,13]. However, a 2021 metagenomic analysis revealed it remained unclear whether individual microbes or microbial signatures consisting of a group of microorganisms are consistently depleted or elevated in OSCC in human patients [24]. This is an active area of current research, and the manipulation of the oral microbiome of people with oral cancer is showing promising results [25].

Despite meaningful advances in our understanding of feline SCC [26], a critical gap remains: the oral bacterial microenvironment in feline OSCC has yet to be comprehensively examined. To the author’s knowledge, no published studies describe the oral microbiota of cats with this cancer, despite OSCC accounting for up to 70% of feline oral neoplasms [27]. Microbiome research in companion animal oral tumors remains sparse, with only a few studies in dogs involving malignant melanoma or mixed-tumor cohorts [18,19,22,28]. Although recent work has examined the skin microbiome in normal and cutaneous SCC-affected cats and dogs [20], comparable investigations of the oral niche in feline OSCC are notably absent. Although there are no other tumor microbiome studies in cats, numerous other feline studies describe the oral microbiome in common oral inflammatory disorders such as feline chronic gingivostomatitis (FCGS) and periodontitis [21].

We hypothesized that the oral microbiome of cats with OSCC differs significantly from that of healthy cats. The objective of this study was to characterize and compare the oral bacterial communities of cats with OSCC and healthy controls using full-length 16S rRNA gene sequencing, and to evaluate differences between tumor-associated and normal oral sites. Given the widespread use of the cat as a comparative model for human OSCC, this study also provides important context for interspecies similarities and differences in oral cancer-associated microbial dysbiosis.

## 2. Materials and Methods

### 2.1. Ethics Statement and Sample Size Consideration

Biofilm sampling consisted of gentle swabbing of oral and tumor surfaces and was performed under general anesthesia concurrently with clinically indicated biopsy or surgical excision procedures. No additional anesthesia, surgical intervention, or tissue disruption was performed for research purposes beyond standard diagnostic or therapeutic care. Tumor tissue was collected as part of a routine biopsy procedure or resulting from tumor excision. This study does not induce disease, injury, or distress in its subjects.

This study was designed as an exploratory, descriptive analysis of the oral microbiota in cats with OSCC. Due to the aggressivity of the disease, ethical constraints, and the requirement that sampling be performed only during clinically indicated procedures under general anesthesia, sample size was determined by case availability during the study period rather than by a priori power calculations. At the time of study initiation, no published data were available to estimate expected effect sizes for feline OSCC-associated oral microbiome differences, precluding reliable formal power analysis.

### 2.2. Client-Owned Animals

Seven client-owned animals (*n* = 7) were recruited from cases presented to the University of California Davis (UC Davis), William R. Pritchard Veterinary Medical Teaching Hospital (VMTH), with a histological diagnosis of oral squamous cell carcinoma, before antibiotic exposure. Swabs, blood and tissue were collected with informed owner consent and UC Davis IACUC Approval (Approval Code: Protocol n. 24338, active protocols are reviewed annually, last approved: 1 May 2025). Twelve healthy client-owned cats served as controls and were recruited from a previous study [21]. Cancer samples were collected by board-certified veterinary dentists and oral surgeons (S. L. G. and M. S. R.) who were responsible for clinical evaluation, diagnosis, and treatment of the oral neoplasms. A full clinicopathologic evaluation included complete blood count, and chemistry panel. Under general anesthesia and prior to any interventions, clinical patients with an oral tumor had their biofilm sampled. Once the tumor tissue was harvested from tumor cases, the intra-tumoral microbiome surface was also sampled.

### 2.3. Sampling Collection

A total of seven cats histopathologically diagnosed with OSCC were included in the study. From each cat, three biofilm samples were obtained using a sterile swab: one from a clinically normal (non-tumor) site, one from the tumor surface, and one from the intra-tumoral surface. For comparison, 12 clinically healthy control cats were sampled in the same manner from the oral mucosa (single site per animal) [21]. Sterile swabs were employed for biofilm collection in the feline oral cavity, a previously validated method [21]. Samples from tumor-bearing cats were collected prior to any oral irrigation, and from non-necrotic portions of the tumor. Immediately after collection, all swabs were labeled individually, placed into separate plastic collection tubes, and stored at −80 °C until shipment to AnimalBiome’s laboratories for analysis.

### 2.4. DNA Extraction, Amplification, and Sequencing

Genomic DNA was extracted using the DNeasy PowerSoil Pro DNA Isolation Kit (Qiagen, Hilden, Germany), following the manufacturer’s instructions. DNA concentration was quantified with the Qubit dsDNA High-Sensitivity Assay Kit (Thermo Fisher Scientific, Waltham, MA, USA). Full-length (V1–V9) 16S rRNA gene amplicons were generated using primers 27F (5′-AGRGTTYGATYMTGGCTCAG-3′) and 1492R (5′-RGYTACCTTGTTACGACTT-3′), tailed with asymmetric barcode sequences in a dual-index one-step PCR. PCR amplification was performed using 12.5 μL of KAPA HiFi HotStart ReadyMix PCR kit (KAPA Biosystems, Wilmington, MA, USA), 3 μL of both Forward and Reverse primers (2.5 μM), 5 μL of template DNA, and PCR-grade water required for a final volume of 25 μL. PCR conditions used were initial denaturation at 95 °C for 3 min and 27 cycles of denaturation at 95 °C for 30 s, annealing at 57 °C for 30 s, and extension at 72 °C for 60 s. Amplicons were pooled into a final library. The purified amplicons were sequenced using PacBio Sequel IIe chemistry (Pacific Biosciences, Menlo Park, CA, USA). Healthy control samples were collected as part of a previously published cohort and were sequenced prior to the OSCC samples [20]. DNA extraction and sequencing were performed using the same laboratory protocols and sequencing platform, reducing technical variability.

### 2.5. Bioinformatic Processing of PacBio CSS Reads

To minimize analytical batch effects, all demultiplexed raw sequence files from healthy controls and OSCC cases were processed together using the same bioinformatic workflow, using the same denoising, filtering, and taxonomic classification settings. Following sequencing, CCS reads were converted to HiFi reads for each demultiplexed sample using SMRT Link (v11.0.0.146107). Read processing—including trimming, denoising, dereplication, and chimera filtering—was carried out in QIIME 2 (v2023.5) [29] using the dada2 plugin [30], following the workflow described in AnimalBiome’s PacBio full-length 16S rRNA tutorial (https://github.com/AnimalBiome/AB_FlexTax/tree/main, accessed on 9 February 2026). In brief, pseudo-pooling was applied during denoising, the maximum expected-error threshold was set to 3, and reads outside the 1300–1600 bp range after adapter removal were discarded. Chimera detection was performed on pooled samples.

For taxonomic annotation, we applied the same Silva v138.1 curation and classification workflow described in our previous publication [21]. Briefly, the Silva NR99 database [31] was filtered to retain primarily full-length prokaryotic 16S rRNA sequences and to remove entries with uninformative species labels, as outlined in the referenced tutorial. ASVs were assigned taxonomy in QIIME 2 using a Naive Bayes classifier (confidence = 0.7) and refined with VSEARCH [32] for higher-resolution matches. Raw PacBio HiFi sequences have been deposited to the NCBI Sequence Read Archive (SRA), under BioProjects PRJNA1023696 (healthy controls) and PRJNA1405963 (OSCC group).

### 2.6. Data and Statistical Analysis

Microbiome data were imported into the R statistical software program (v.4.3.0) for subsequent analysis. For alpha- and beta-diversity analyses, the data were further processed using the R package MicrobiomeAnalystR (Xia Lab, McGill University, Montreal, QC, Canada) [33]. A low-count filter was used to filter all features with <4 counts in at least 20% of the values. Features with <10% variance, based on the inter-quartile rank, were filtered using a low-variance filter. Finally, for data scaling, the total sum scaling was applied at the bacterial genus level. Alpha diversity analyses were performed using the Shannon index and Kruskal–Wallis nonparametric test, whereas beta-diversity analyses were performed using the Bray–Curtis dissimilarity index and PERMANOVA tests [34]. Furthermore, we looked at the microbial composition of each sample, and we compared across different groups and different sample sites. Additionally, visualization of the microbial composition has been performed through a heatmap, in order to highlight correlations between taxa and the different groups. Microbial associations with other clinical factors, as shown in Table 1, were not possible due to the low patient sample size.

## 3. Results

To determine whether the oral microbiota of cats with OSCC differ significantly from healthy cats, we sampled 19 cats: 12 healthy controls and 7 cats with OSCC (Table 1). Based on the information collected upon clinical visit, OSCC cats were generally older than healthy cats (median age 14 vs. 7 years). The proportion of neutered males was similar between groups (43% vs. 58%). One cat with OSCC had feline chronic gingivostomatitis (FCGS); otherwise there was no concurrent oral or systemic disease in either cohort.

### 3.1. Overall Community Structure Differs Between Healthy Cats and Those with OSCC

**Figure 1:**
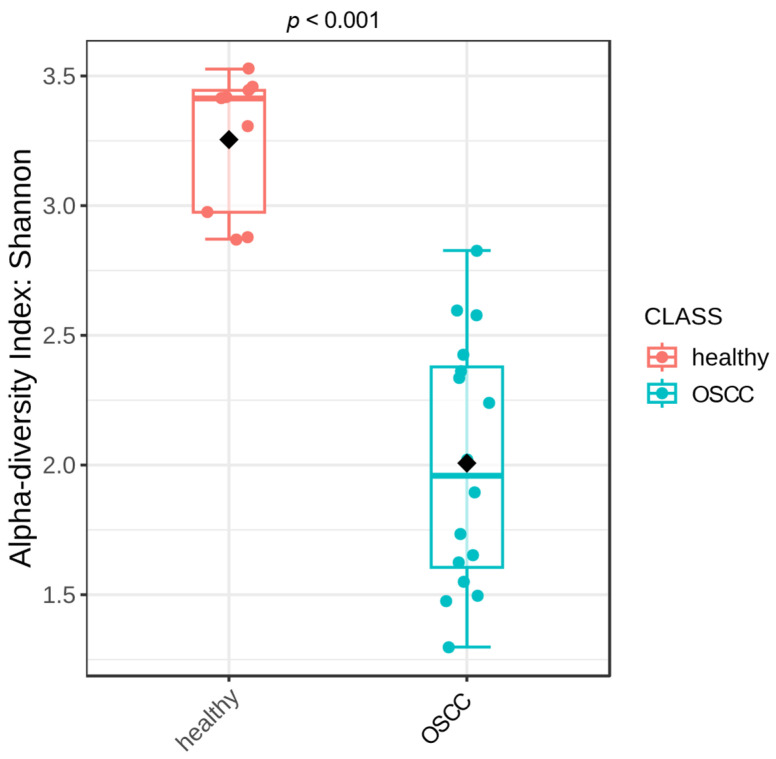
Oral microbiota alpha diversity of healthy cats compared to cats with oral squamous cell carcinoma. For cats with OSCC, samples from the tumor surface and tumor tissue were combined. Individual samples are shown as colored points, whereas the black diamond denotes the group centroid (average community position in ordination space).

**Figure 2:**
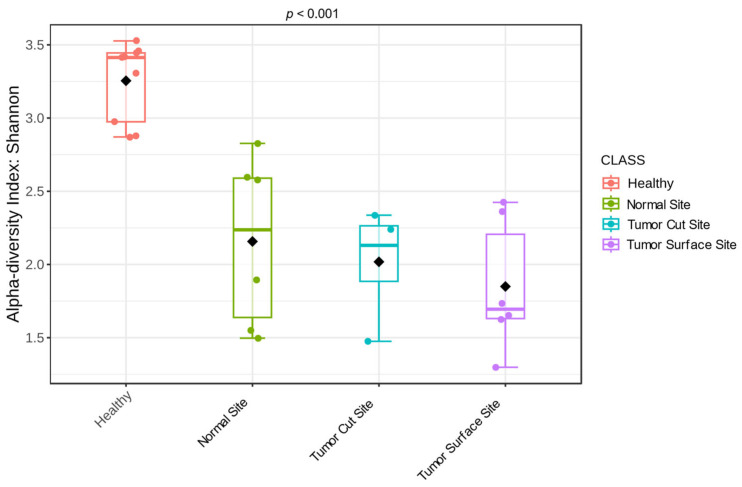
Oral microbiota alpha diversity by subsite in cats with OSCC. Diversity differed between healthy cats and the OSCC subsites, but not between subsites. Individual samples are shown as colored points, whereas the black diamond denotes the group centroid (average community position in ordination space).

Shannon diversity was significantly lower in OSCC samples compared with healthy controls (Figure 1; Kruskal–Wallis test, *p* < 0.001). The three subgroups of OSCC samples, normal mucosa, tumor surface, and intra-tumoral cut surface, remained significantly less diverse than healthy samples (*p* < 0.001). No significant differences in alpha diversity were detected among the OSCC subsites (Figure 2).

**Figure 3:**
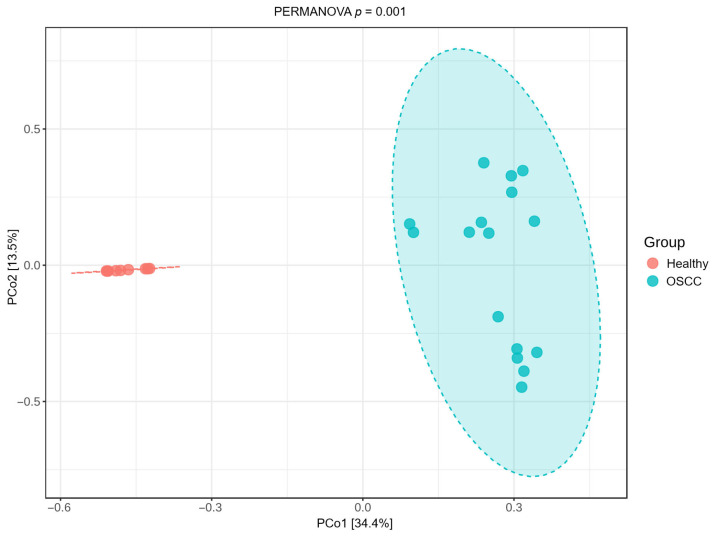
Beta diversity of the feline oral microbiome in health and oral squamous cell carcinoma (OSCC). Principal Coordinate Analysis (PCoA) of Bray–Curtis dissimilarity demonstrates significant differences in overall microbial community composition between healthy cats and cats with OSCC (PERMANOVA, *p* = 0.001).

Principal Coordinate Analysis (PCoA) based on Bray–Curtis dissimilarity showed a clear separation between healthy cats and OSCC cases. Healthy samples formed a tight cluster, while OSCC samples were more dispersed (Figure 3). PERMANOVA indicated a significant difference in beta diversity between groups (*p* = 0.001).

**Figure 4:**
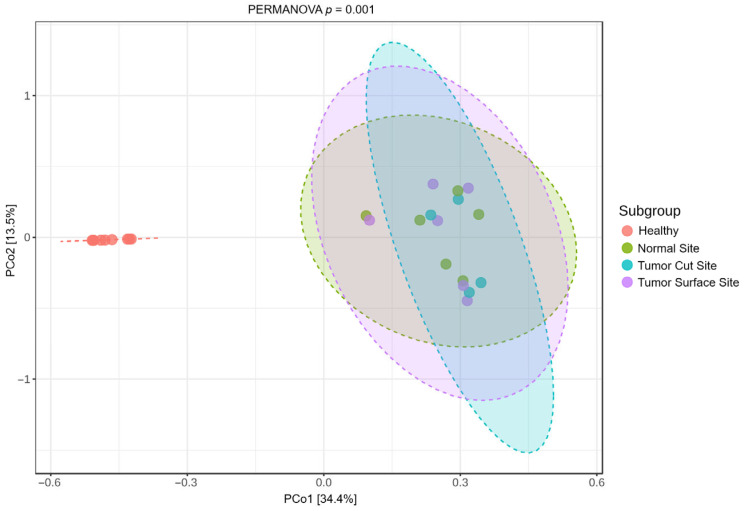
Beta diversity of the feline oral microbiome stratified by OSCC sampling subsite. Bray–Curtis-based PCoA demonstrates significant separation between healthy oral microbiomes and OSCC-associated microbiomes (PERMANOVA, *p* = 0.001). Stratification of OSCC samples by collection site does not reveal clear subsite-specific clustering, with overlapping community composition observed across tumor-associated sites.

Beta-diversity patterns were consistent across OSCC subsites. All OSCC subgroups—normal mucosa, tumor cut site, and tumor surface—were significantly different from healthy controls (PERMANOVA, *p* ≤ 0.003), but the OSCC sample sites did not differ significantly from each other (*p* > 0.3; Figure 4). Collectively, these findings indicate that individual samples are dominated by a few taxa and that different samples can be dominated by entirely different taxa. Additionally, OSCC-associated microbial shifts occur across the tumor environment rather than being restricted to a specific tissue region.

### 3.2. Taxonomic Differences Between Healthy and OSCC Oral Microbiomes

**Figure 5:**
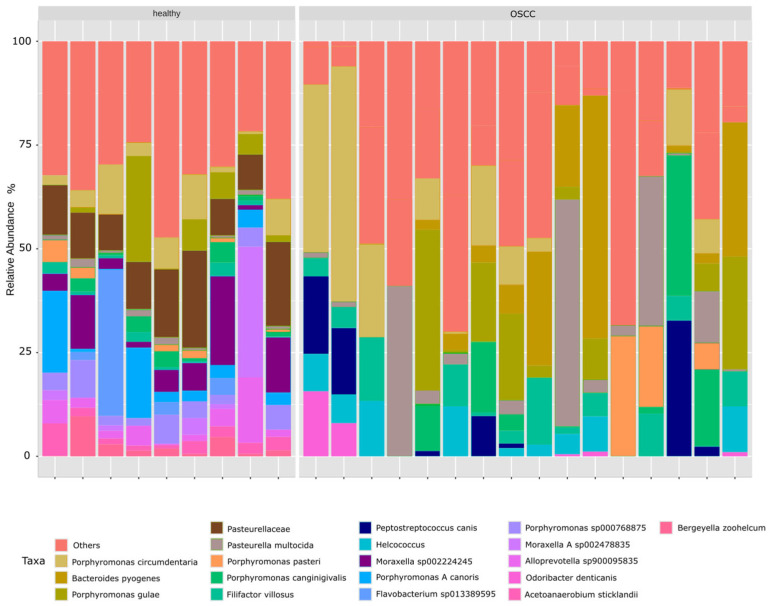
Taxonomic composition of the feline oral microbiome in healthy cats and cats with oral squamous cell carcinoma (OSCC). Relative abundance of dominant oral bacterial taxa in healthy cats and cats with OSCC. Each bar represents an individual sample, with colors indicating taxa. Taxa present at low relative abundance are grouped as “Others”.

**Figure 6:**
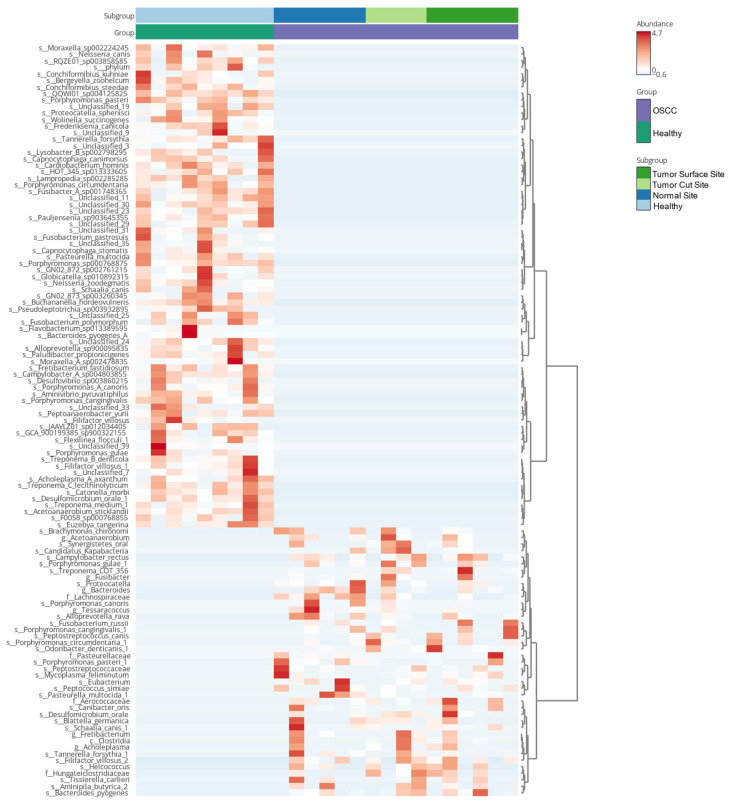
Clustering heatmap of differentially abundant oral bacterial taxa in healthy cats and cats with OSCC. Heatmap of normalized relative abundances of selected oral bacterial taxa across healthy and OSCC samples. Samples are annotated by disease status and OSCC sampling subsite. Taxa and samples are hierarchically clustered based on abundance profiles.

Relative abundance profiles showed pronounced shifts in microbial composition between groups (Figure 5). Healthy cats’ core microbiome was primarily characterized by species such as *Porphyromonas canoris*, *Porphyromonas cangingivalis*, *Porphyromonas pasteri*, and *Moraxella* spp., which dominated the community structure. In contrast, the variable microbiome in OSCC samples exhibited increased abundances of several anaerobic and disease-associated taxa, including *Filifactor villosus*, *Bacteroides pyogenes*, *Odoribacter denticanis*, *Porphyromonas circumdentaria*, and members of the Pasteurellaceae. These differences were further supported by the heatmap of dominant species (Figure 6), which highlighted a more heterogeneous and dysbiotic microbial profile in cats with OSCC relative to the consistent commensal composition observed in healthy controls.

### 3.3. Inferred Ecological Implications of Oral Microbial Dysbiosis in Feline OSCC Compared with Human Disease

**Figure 7:**
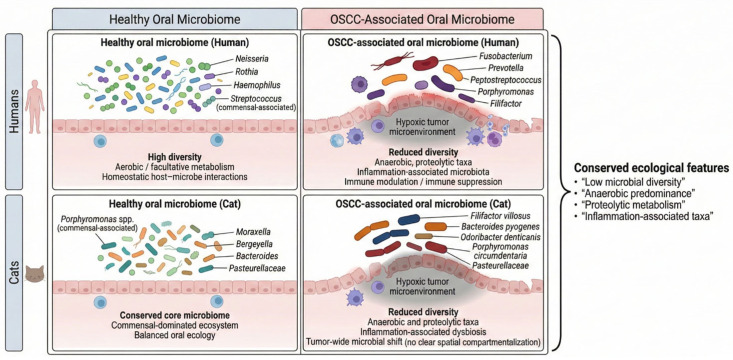
Schematic representation of taxonomic and inferred ecological differences between health and oral squamous cell carcinoma (OSCC) in human and feline hosts.

Because this study used 16S rRNA gene amplicon sequencing, functional pathways cannot be directly assessed; therefore, the following interpretations are speculative and based on known ecological associations of the taxa observed. Compared with control oral samples, feline OSCC was characterized by a marked restructuring of the oral microbial community. Tumor-associated samples exhibited reduced alpha diversity and a shift in taxonomic composition toward organisms commonly associated with anaerobic or inflammation-linked oral niches. Specifically, feline OSCC samples showed increased relative abundance of *Filifactor villosus*, *Bacteroides pyogenes*, *Odoribacter denticanis*, *Porphyromonas circumdentaria*, and members of the Pasteurellaceae, alongside depletion of taxa frequently associated with oral health, including *Moraxella*- and *Bergeyella*-like organisms. While the specific taxa enriched in feline OSCC differed from those commonly reported in human OSCC, the overall ecological pattern—characterized by reduced diversity and enrichment of anaerobe-associated taxa—was consistent with trends reported in human oral squamous cell carcinoma. These taxonomic and diversity shifts form the basis for a comparative conceptual model of conserved ecological restructuring in OSCC across cats and humans (Figure 7).

## 4. Discussion

This study provides the first description of the oral microbiota associated with feline oral squamous cell carcinoma (OSCC) and its preliminary comparison with clinically healthy cats. Given the high prevalence and poor prognosis of OSCC in cats, the absence of prior microbiome data has represented a significant knowledge gap. Our results show that feline OSCC is probably associated with significant oral microbial dysbiosis, characterized by reduced alpha diversity, distinct beta-diversity clustering, and consistent taxonomic shifts relative to healthy controls. These findings support our hypothesis that the oral microbiome of cats with OSCC differs significantly from that of healthy cats and establish a foundation for future needed mechanistic and translational studies [35,36]. Due to the limited number of cats enrolled in this study, larger studies with matched confounding factors will be needed in the future.

Healthy cats displayed a relatively conserved oral core microbiome dominated by *Porphyromonas* spp., *Moraxella* spp., *Bacteroides*, Pasteurellaceae, *Helcococcus*, and *Moraxella*-like taxa, consistent with previous feline oral microbiome studies [21,37,38,39,40,41,42,43]. In contrast, OSCC samples exhibited increased abundances of anaerobic, proteolytic, and disease-associated taxa, including *Filifactor villosus*, *Bacteroides pyogenes*, *Odoribacter denticanis*, *Porphyromonas circumdentaria*, and members of the Pasteurellaceae, alongside depletion of putative health-associated commensals such as *Moraxella*- and *Bergeyella*-like taxa. These shifts reflect a transition from a relatively stable, commensal-rich microbiome toward a low-diversity, anaerobe-dominated community. Such a pattern is consistent with microbial dysbiosis observed in chronic inflammatory and neoplastic oral diseases across species and suggests that functional ecological changes—rather than the presence of a single pathogenic organism—may be relevant to disease processes in feline OSCC [22,44,45].

Although this study was based on full-length 16S rRNA gene sequencing and therefore does not directly assess microbial gene expression or metabolic activity, the observed taxonomic shifts could be interpreted within a broader ecological framework informed by human OSCC microbiome studies. In humans, OSCC is consistently associated with enrichment of anaerobic, proteolytic, and inflammation-linked taxa, including *Porphyromonas*, *Fusobacterium*, *Peptostreptococcus*, *Prevotella*, and *Filifactor*, accompanied by depletion of health-associated commensals such as *Neisseria*, *Haemophilus*, and *Rothia*. Our findings reveal a concordant ecological shift in cats despite differences in specific taxa, suggesting that conservation is strongest at the level of microbial guilds and ecological behavior rather than species-level identity. Notably, *Fusobacterium nucleatum*, a prominent organism in human OSCC [46,47], was not a dominant feature of feline OSCC samples. This difference likely reflects host-specific oral ecology rather than methodological limitations. Thus, while cats are often considered a comparative model for human HNSCC [2], our results emphasize that directional microbiome changes and functional associations may be more conserved than individual taxa.

No significant microbiome differences were detected among samples collected from tumor surfaces, tumor cut surfaces, or clinically normal mucosa within OSCC-affected cats. However, this finding should be interpreted cautiously, as the absence of detectable subsite differences may reflect limited statistical power and/or the constraints of swab-based sampling rather than true biological homogeneity. While our results are consistent with the possibility that OSCC-associated microbial alterations extend beyond the visible tumor mass and may involve the broader oral environment, additional studies with larger cohorts and higher spatial resolution will be required to confirm whether a “field effect” is present in feline OSCC. This is different from humans, as studies using spatial transcriptomics, multiplex imaging, and 3D high-resolution mapping across multiple human cancers (including oral/head and neck tumors) have shown that bacteria are not randomly distributed but are localized within specific tumor niches. The tumor surface in these studies was enriched with periodontal-associated anaerobes such as *Fusobacterium* and *Prevotella*, the hypoxic tumor core harbored immune-modulating taxa, and the adjacent mucosa resembled healthy microbiota, with these spatially distinct communities closely correlating with tumor pathology and clinical behavior. Consequently, intra-tumoral bacteria may actively shape cellular heterogeneity, immune exclusion, and tumor invasiveness [48,49]. Several non-mutually exclusive explanations could possibly explain this difference in cats. First, the small size of cat teeth, the more conical shape, and the lack of interproximal contacts in feline teeth differ considerably from human dentition. Secondly, a field effect may exist in which tumor-associated ecological changes influence adjacent mucosal sites. Eventually, dysbiosis may precede tumor development, resulting in a relatively uniform microbial profile across oral sites at the time of diagnosis. Third, sampling limitations and sample size may have reduced the ability to detect subtle spatial differences. Regardless, these findings contrast with some human OSCC studies in which tumor tissue and adjacent normal mucosa differ microbiologically, highlighting hypothetical potential species-specific differences in oral tumor biology and disease progression [50,51].

Microbiome studies in dogs with oral tumors have yielded mixed results. Some investigations have identified significant dysbiosis and enrichment of periodontal-associated taxa [18,28], whereas others report only minor differences compared to healthy controls [22]. Our findings in cats more closely resemble studies demonstrating tumor-associated dysbiosis, suggesting that microbial involvement may vary by species, tumor type, or oral microenvironment. These interspecies differences caution against direct extrapolation of microbiome signatures across companion animals and reinforce the importance of species-specific investigations. A descriptive evaluation of the oral microbiota in canine OSCC is forthcoming from our group.

In cats with severe inflammatory oral diseases like feline chronic gingivostomatitis [21], the oral microbiota across ten sites displayed similar patterns of dysbiosis as those seen in cats with oral cancer, with several taxa overlapping between the two. The oral communities of FCGS cats also have a depletion of healthy-associated bacteria such as *Moraxella*, *Neisseria*, *Conchiformibius kuhniae*, *Bergeyella zoohelcum*, and *Porphyromonas pasteri*, and elevated abundances of taxa such as *Porphyromonas gulae*, *P. circumdentaria*, *Bacteroides pyogenes*, *Tanerella forsythia*, and *Fusobacterium russii*. This suggests that microbial dysbiosis characterized by depletion of healthy-associated bacteria throughout the oral cavity is one of the hallmarks of oral disease in cats. Whether the observed dysbiosis represents a predisposing factor, a consequence of chronic inflammation, or a tumor-driven ecological shift remains unknown. However, the present study was not designed to assess causality or temporal relationships. The shared dysbiotic signatures observed in OSCC and FCGS raise the possibility of a linkage between chronic inflammation and oral carcinogenesis. Epidemiologic observations indicate that chronic oral inflammation may precede OSCC development in some cats [52], raising the possibility of shared microbial and immunologic pathways. However, retrospective data from veterinary teaching hospitals did not show a positive association between refractory FCGS and oral SCC; in one cohort, the presence of FCGS was inversely associated with SCC occurrence, suggesting a lower rate of SCC in FCGS cats [53]. Longitudinal studies tracking cats with inflammatory oral disease over time will be required to clarify these and many other relationships.

It is tempting to borrow information from the human literature on potential mechanisms of disease and crosstalk between inflammation and the immune environment of companion animal squamous cell carcinoma. However, cats and dogs are not small humans, and many unique differences exist. Probably the most overt distinction is the shortened life span of dogs and cats. As such, tumor progression in the oral cavity is rapid and generally lacks overt epithelial dysplasia and oral potentially malignant disease (OPMD). OPMD is common in people and indicates which lesions need to be followed for malignant transformation. OPMD may be present for years in people with oral lichen planus, and in areas of erythroplakia or leukoplakia and then suddenly transform to OSCC [54]. While feline OSCC is often considered a comparative model for human disease, our observations should be interpreted as descriptive and hypothesis-generating; functional studies will be required to determine whether similar microbial activities or host–microbe mechanisms are conserved across species.

Emerging evidence in human OSCC suggests that anaerobic bacterial predominance within tumors may influence the immune microenvironment, including T-cell dysfunction and immune suppression [55]. Preliminary parallel data in feline OSCC indicate alterations in regulatory T-cell populations, raising the possibility of microbiome–immune interactions in this disease [2].

Although direct correlations between immune cell subsets and microbial taxa were beyond the scope of this study, future integrative analyses may provide insights into the mechanisms linking oral dysbiosis, immune modulation, and tumor progression in cats.

This study has several limitations. The limited sample size, particularly within the OSCC cohort, represents an important limitation of this study and restricts statistical power as well as the ability to assess associations with clinical variables. In addition, the OSCC cohort was older than the healthy controls, and age may influence microbiome composition, representing a potential confounder when comparing groups. While age-related microbiome differences have been documented in cats at other body sites such as the intestinal microbiota [56,57], evidence for age-associated shifts specifically in the feline oral microbiome remains limited [58]. Future studies should therefore include age-matched control cats to more clearly define OSCC-specific microbial changes. Recruitment was additionally limited by the difficulty of sampling tumor-bearing cats prior to antibiotic exposure, as many OSCC patients receive antimicrobials before referral due to oral infection/necrosis and clinical discomfort. To minimize antibiotic-driven bias in microbial community composition, we prioritized pre-antibiotic sampling, which further restricted case availability and reduced the final cohort size.

Finally, one OSCC cat had concurrent feline chronic gingivostomatitis (FCGS), an inflammatory oral disease likely to independently alter the oral microbiota and increase variability within the OSCC group. Larger prospective studies with complete metadata and exclusion of concurrent inflammatory oral disease will be necessary to confirm OSCC-specific microbial signatures.

Viral cofactors, including papillomavirus, FeLV, and FIV, were not evaluated and may influence both tumor biology and microbial composition. The use of oral mucosal and tumor surface swabs provided a standardized approach for sampling across cats and sites; however, more localized sampling may be needed to detect fine-scale microbial differences between the tumor sites. Futhermore, tumors were located in different areas of the mouth. Additionally, while full-length 16S rRNA sequencing provides high taxonomic resolution, it does not yield functional information. Metagenomic, metatranscriptomic, and metabolomic approaches will be necessary to define functional pathways relevant to feline OSCC. Accordingly, these findings should be interpreted as preliminary and hypothesis-generating.

## 5. Conclusions

Collectively, these findings hypothetically demonstrate that feline OSCC is associated with a distinct oral microbial ecosystem characterized by reduced diversity and enrichment of anaerobic, disease-associated taxa. While specific bacterial species differ between cats and humans, the overall ecological shift toward dysbiosis appears conserved. This study is limited by a small and unbalanced cohort, age differences between groups, and unavoidable confounders inherent to studying feline oral squamous cell carcinoma under ethical and clinical constraints. These limitations restrict the strength of inferential conclusions as exploratory, descriptive, and hypothesis-generating. Although there are many limitations, this study provides a critical baseline for future investigations into diagnostic biomarkers, immune interactions, and microbiome-targeted interventions in feline oral cancer.

## Figures and Tables

**Table 1:** Median age, sex, and body weight of healthy and cancer cats affected with oral squamous cell carcinoma (OSCC).

	Healthy (*n* = 12)	OSCC (*n* = 7)
Age (years)	7.6	14.8
Sex (% male)	58	43
Body weight (kg)	4.5	4

## Data Availability

Raw PacBio HiFi sequences have been deposited to the NCBI Sequence Read Archive (SRA), under BioProjects PRJNA1023696 (healthy controls) and PRJNA1405963 (OSCC group).

## Abbreviations

The following abbreviations are used in this manuscript:

OSCC	Oral Squamous Cell Carcinoma
HNSCC	Head and Neck Squamous Cell Carcinoma
FeLV	Feline Leukemia Virus
FIV	Feline Immunodeficiency Virus
PV	Papilloma Virus
OPMD	Oral Potentially Malignant Disease

## References

[1] Cray M., Selmic L.E., Ruple A. (2020). Demographics of Dogs and Cats with Oral Tumors Presenting to Teaching Hospitals: 1996–2017. J. Vet. Sci..

[2] Sparger E.E., Murphy B.G., Kamal F.M., Arzi B., Naydan D., Skouritakis C.T., Cox D.P., Skorupski K. (2018). Investigation of Immune Cell Markers in Feline Oral Squamous Cell Carcinoma. Vet. Immunol. Immunopathol..

[3] Sukmana B.I., Saleh R.O., Najim M.A., AL-Ghamdi H.S., Achmad H., Al-Hamdani M.M., Taher A.A., Alsalamy A., Khaledi M., Javadi K. (2024). Oral Microbiota and Oral Squamous Cell Carcinoma: A Review of Their Relation and Carcinogenic Mechanisms. Front. Oncol..

[4] Fitzsimonds Z.R., Rodriguez-Hernandez C.J., Bagaitkar J., Lamont R.J. (2020). From Beyond the Pale to the Pale Riders: The Emerging Association of Bacteria with Oral Cancer. J. Dent. Res..

[5] Shin J.M., Kamarajan P., Fenno J.C., Rickard A.H., Kapila Y.L. (2016). Metabolomics of Head and Neck Cancer: A Mini-Review. Front. Physiol..

[6] Rajeev R., Choudhary K., Panda S., Gandhi N. (2012). Role of Bacteria in Oral Carcinogenesis. South Asian J. Cancer.

[7] Fonsêca T.C., Jural L.A., Marañón-Vásquez G.A., Magno M.B., Roza A.L.O.C., Ferreira D.M.T.P., Maia L.C., Romañach M.J., Agostini M., Abrahão A.C. (2023). Global Prevalence of Human Papillomavirus-Related Oral and Oropharyngeal Squamous Cell Carcinomas: A Systematic Review and Meta-Analysis. Clin. Oral Investig..

[8] Khoshbayan A., Narimisa N., Razavi S., Shariati A., Tavakoli-Yaraki M., Darban-Sarokhalil D., Emami Razavi A. (2025). The Interactions of Fusobacterium Nucleatum and Porphyromonas Gingivalis with microRNAs: Contributions to Oral Squamous Cell Carcinoma. Mol. Biol. Rep..

[9] Świątkowski W., Bakiera A., Rahnama-Hezavah M., Grywalska E., Niedzielski A., Korona-Głowniak I. (2025). The Oral Microbiota Change in Oral Cancer—A Preliminary Study. Ann. Agric. Environ. Med..

[10] Lin D., Yang L., Wen L., Lu H., Chen Q., Wang Z. (2021). Crosstalk between the Oral Microbiota, Mucosal Immunity, and the Epithelial Barrier Regulates Oral Mucosal Disease Pathogenesis. Mucosal Immunol..

[11] Yang C.-Y., Yeh Y.-M., Yu H.-Y., Chin C.-Y., Hsu C.-W., Liu H., Huang P.-J., Hu S.-N., Liao C.-T., Chang K.-P. (2018). Oral Microbiota Community Dynamics Associated With Oral Squamous Cell Carcinoma Staging. Front. Microbiol..

[12] Karpiński T.M. (2019). Role of Oral Microbiota in Cancer Development. Microorganisms.

[13] Ting H.S.L., Chen Z., Chan J.Y.K. (2023). Systematic Review on Oral Microbial Dysbiosis and Its Clinical Associations with Head and Neck Squamous Cell Carcinoma. Head Neck.

[14] Deo P.N., Deshmukh R. (2019). Oral Microbiome: Unveiling the Fundamentals. J. Oral Maxillofac. Pathol..

[15] Turnbaugh P.J., Ley R.E., Hamady M., Fraser-Liggett C.M., Knight R., Gordon J.I. (2007). The Human Microbiome Project. Nature.

[16] Zarco M., Vess T., Ginsburg G. (2012). The Oral Microbiome in Health and Disease and the Potential Impact on Personalized Dental Medicine. Oral Dis..

[17] Petersen C., Round J.L. (2014). Defining Dysbiosis and Its Influence on Host Immunity and Disease. Cell Microbiol..

[18] Pinto C., Aluai-Cunha C., Santos A. (2023). The Human and Animals’ Malignant Melanoma: Comparative Tumor Models and the Role of Microbiome in Dogs and Humans. Melanoma Res..

[19] Santiago-Rodriguez T.M. (2024). Comparative Oncology Using Domesticated Dogs and Their Microbiome. Front. Vet. Sci..

[20] Bromfield J.I., Zaugg J., Straw R.C., Cathie J., Krueger A., Sinha D., Chandra J., Hugenholtz P., Frazer I.H. (2024). Characterization of the Skin Microbiome in Normal and Cutaneous Squamous Cell Carcinoma Affected Cats and Dogs. mSphere.

[21] Anderson J.G., Rojas C.A., Scarsella E., Entrolezo Z., Jospin G., Hoffman S.L., Force J., MacLellan R.H., Peak M., Shope B.H. (2023). The Oral Microbiome across Oral Sites in Cats with Chronic Gingivostomatitis, Periodontal Disease, and Tooth Resorption Compared with Healthy Cats. Animals.

[22] Lisjak A., Lopes B.C., Pilla R., Nemec A., Suchodolski J.S., Tozon N., Lisjak A., Lopes B.C., Pilla R., Nemec A. (2023). A Comparison of the Oral Microbiota in Healthy Dogs and Dogs with Oral Tumors. Animals.

[23] Mager D., Haffajee A., Devlin P., Norris C., Posner M., Goodson J. (2005). The Salivary Microbiota as a Diagnostic Indicator of Oral Cancer: A Descriptive, Non-Randomized Study of Cancer-Free and Oral Squamous Cell Carcinoma Subjects. J. Transl. Med..

[24] Mun L.S., Lum S.W., Sze G.K.Y., Yoong C.H., Yung K.C., Lok L.K., Gopinath D., Mun L.S., Lum S.W., Sze G.K.Y. (2021). Association of Microbiome with Oral Squamous Cell Carcinoma: A Systematic Review of the Metagenomic Studies. Int. J. Environ. Res. Public Health.

[25] Mivehchi H., Eskandari-Yaghbastlo A., Pour Bahrami P., Elhami A., Faghihinia F., Nejati S.T., Kazemi K.S., Nabi Afjadi M. (2025). Exploring the Role of Oral Bacteria in Oral Cancer: A Narrative Review. Discov. Oncol..

[26] Tutu P., Daraban Bocaneti F., Altamura G., Dascalu M.A., Horodincu L., Soreanu O.D., Tanase O.I., Borzacchiello G., Mares M. (2025). Feline Oral Squamous Cell Carcinoma: Recent Advances and Future Perspectives. Front. Vet. Sci..

[27] Cotter S.M. (1981). Oral Pharyngeal Neoplasms in the Cat. J. Am. Anim. Hosp. Assoc..

[28] Anderson J.G., Paster B.J., Kokaras A., Chen T. (2021). Characterization of the Oral Microbiome in Canine Chronic Ulcerative Stomatitis. J. Immunol. Res..

[29] Bolyen E., Rideout J.R., Dillon M.R., Bokulich N.A., Abnet C.C., Al-Ghalith G.A., Alexander H., Alm E.J., Arumugam M., Asnicar F. (2019). Reproducible, Interactive, Scalable and Extensible Microbiome Data Science Using QIIME 2. Nat. Biotechnol..

[30] Callahan B.J., McMurdie P.J., Rosen M.J., Han A.W., Johnson A.J.A., Holmes S.P. (2016). DADA2: High-Resolution Sample Inference from Illumina Amplicon Data. Nat. Methods.

[31] Quast C., Pruesse E., Yilmaz P., Gerken J., Schweer T., Yarza P., Peplies J., Glöckner F.O. (2013). The SILVA Ribosomal RNA Gene Database Project: Improved Data Processing and Web-Based Tools. Nucleic Acids Res..

[32] Rognes T., Flouri T., Nichols B., Quince C., Mahé F. (2016). VSEARCH: A Versatile Open Source Tool for Metagenomics. PeerJ.

[33] Chong J., Liu P., Zhou G., Xia J. (2020). Using MicrobiomeAnalyst for Comprehensive Statistical, Functional, and Meta-Analysis of Microbiome Data. Nat. Protoc..

[34] Oksanen J., Simpson G.L., Blanchet F.G., Kindt R., Legendre P., Minchin P.R., O’Hara R.B., Solymos P., Stevens M.H.H., Szoecs E. (2025). Vegan: Community Ecology Package, version 2.7-2. https://cran.r-project.org/web/packages/vegan/index.html.

[35] Tannehill-Gregg S.H., Levine A.L., Rosol T.J. (2006). Feline Head and Neck Squamous Cell Carcinoma: A Natural Model for the Human Disease and Development of a Mouse Model. Vet. Comp. Oncol..

[36] Cannon C.M. (2015). Cats, Cancer and Comparative Oncology. Vet. Sci..

[37] Adler C.J., Malik R., Browne G.V., Norris J.M. (2016). Diet May Influence the Oral Microbiome Composition in Cats. Microbiome.

[38] Older C.E., Diesel A.B., Lawhon S.D., Queiroz C.R.R., Henker L.C., Hoffmann A.R. (2019). The Feline Cutaneous and Oral Microbiota Are Influenced by Breed and Environment. PLoS ONE.

[39] Sturgeon A., Pinder S.L., Costa M.C., Weese J.S. (2014). Characterization of the Oral Microbiota of Healthy Cats Using Next-Generation Sequencing. Vet. J..

[40] Dewhirst F.E., Klein E.A., Bennett M.-L., Croft J.M., Harris S.J., Marshall-Jones Z.V. (2015). The Feline Oral Microbiome: A Provisional 16S rRNA Gene Based Taxonomy with Full-Length Reference Sequences. Vet. Microbiol..

[41] Harris S., Croft J., O’Flynn C., Deusch O., Colyer A., Allsopp J., Milella L., Davis I.J. (2015). A Pyrosequencing Investigation of Differences in the Feline Subgingival Microbiota in Health, Gingivitis and Mild Periodontitis. PLoS ONE.

[42] Rodrigues M.X., Bicalho R.C., Fiani N., Lima S.F., Peralta S. (2019). The Subgingival Microbial Community of Feline Periodontitis and Gingivostomatitis: Characterization and Comparison between Diseased and Healthy Cats. Sci. Rep..

[43] Weese S.J., Nichols J., Jalali M., Litster A. (2015). The Oral and Conjunctival Microbiotas in Cats with and without Feline Immunodeficiency Virus Infection. Vet. Res..

[44] Setthawongsin C., Khunbutsri D., Pisamai S., Raksajit W., Ngamkala S., Jarudecha T., Meekhanon N., Rungsipipat A. (2023). Isolation of Oral Bacteria, Measurement of the C-Reactive Protein, and Blood Clinical Parameters in Dogs with Oral Tumor. Vet. Med. Int..

[45] Nejman D., Livyatan I., Fuks G., Gavert N., Zwang Y., Geller L.T., Rotter-Maskowitz A., Weiser R., Mallel G., Gigi E. (2020). The Human Tumor Microbiome Is Composed of Tumor Type–Specific Intracellular Bacteria. Science.

[46] Chang C., Geng F., Shi X., Li Y., Zhang X., Zhao X., Pan Y. (2019). The Prevalence Rate of Periodontal Pathogens and Its Association with Oral Squamous Cell Carcinoma. Appl. Microbiol. Biotechnol..

[47] Al-hebshi N.N., Nasher A.T., Maryoud M.Y., Homeida H.E., Chen T., Idris A.M., Johnson N.W. (2017). Inflammatory Bacteriome Featuring Fusobacterium Nucleatum and Pseudomonas Aeruginosa Identified in Association with Oral Squamous Cell Carcinoma. Sci. Rep..

[48] Galeano Niño J.L., Wu H., LaCourse K.D., Kempchinsky A.G., Baryiames A., Barber B., Futran N., Houlton J., Sather C., Sicinska E. (2022). Effect of the Intratumoral Microbiota on Spatial and Cellular Heterogeneity in Cancer. Nature.

[49] Zeng B., Tan J., Guo G., Li Z., Yang L., Lao X., Wang D., Ma J., Zhang S., Liao G. (2022). The Oral Cancer Microbiome Contains Tumor Space–Specific and Clinicopathology-Specific Bacteria. Front. Cell. Infect. Microbiol..

[50] Mandal D.P., Mohanty N., Behera P.K., Gopinath D., Panda S., Al-Kheraif A.A., Divakar D.D., Anil S., Panda S. (2021). A Plausible Proposition of CCL20-Related Mechanism in Fusobacterium Nucleatum-Associated Oral Carcinogenesis. Life.

[51] Zhang L., Liu Y., Zheng H.J., Zhang C.P. (2020). The Oral Microbiota May Have Influence on Oral Cancer. Front. Cell. Infect. Microbiol..

[52] Morrison W.B. (2012). Inflammation and Cancer: A Comparative View. J. Vet. Intern. Med..

[53] Tsugawa A.J., Soltero-Rivera M.M., Goldschmidt S., Arzi B., Kell T., Hoyer N., Bell C.M., Gao H., Shan G., Vapniarsky N. (2025). Co-Occurrence of Feline Chronic Gingivostomatitis and Oral Squamous Cell Carcinoma in 4 Cats (2014–2024). Front. Vet. Sci..

[54] Iocca O., Sollecito T.P., Alawi F., Weinstein G.S., Newman J.G., De Virgilio A., Di Maio P., Spriano G., Pardiñas López S., Shanti R.M. (2020). Potentially Malignant Disorders of the Oral Cavity and Oral Dysplasia: A Systematic Review and Meta-Analysis of Malignant Transformation Rate by Subtype. Head Neck.

[55] Kashima K., Saito T., Kajikawa H., Kawashima A., Ueyama A., Uzawa N., Wada H. (2025). Impact of Oral Anaerobic Bacteria on the Tumor Immune Microenvironment and Prognosis of Oral Cancer. J. Transl. Med..

[56] Masuoka H., Shimada K., Kiyosue-Yasuda T., Kiyosue M., Oishi Y., Kimura S., Ohashi Y., Fujisawa T., Hotta K., Yamada A. (2017). Transition of the Intestinal Microbiota of Cats with Age. PLoS ONE.

[57] Tian T., Zhou Y., Xu Y., Xu Y. (2023). Intestinal Microbial 16S Sequencing and LC-MS Metabonomic Analysis Revealed Differences between Young and Old Cats. Heliyon.

[58] Dorn E.S., Tress B., Suchodolski J.S., Nisar T., Ravindran P., Weber K., Hartmann K., Schulz B.S. (2017). Bacterial Microbiome in the Nose of Healthy Cats and in Cats with Nasal Disease. PLoS ONE.

